# Paradise by the far-red light: Far-red and red:blue ratios independently affect yield, pigments, and carbohydrate production in lettuce, *Lactuca sativa*

**DOI:** 10.3389/fpls.2024.1383100

**Published:** 2024-04-30

**Authors:** Jordan B. Van Brenk, Sarah Courbier, Celestin L. Kleijweg, Julian C. Verdonk, Leo F. M. Marcelis

**Affiliations:** ^1^ Horticulture and Product Physiology, Plant Sciences Group, Wageningen University and Research, Wageningen, Netherlands; ^2^ Faculty of Biology II, University of Freiburg, Freiburg, Germany; ^3^ Centre for Integrative Biological Signalling Studies (CIBSS), University of Freiburg, Freiburg, Germany

**Keywords:** controlled environment agriculture, light quality, far-red light, red:blue ratio, nutritional quality, metabolic compounds, product physiology

## Abstract

In controlled environment agriculture, customized light treatments using light-emitting diodes are crucial to improving crop yield and quality. Red (R; 600-700 nm) and blue light (B; 400-500 nm) are two major parts of photosynthetically active radiation (PAR), often preferred in crop production. Far-red radiation (FR; 700-800 nm), although not part of PAR, can also affect photosynthesis and can have profound effects on a range of morphological and physiological processes. However, interactions between different red and blue light ratios (R:B) and FR on promoting yield and nutritionally relevant compounds in crops remain unknown. Here, lettuce was grown at 200 µmol m^-2^ s^-1^ PAR under three different R:B ratios: R:B_87.5:12.5_ (12.5% blue), R:B_75:25_ (25% blue), and R:B_60:40_ (40% blue) without FR. Each treatment was also performed with supplementary FR (50 µmol m^-2^ s^-1^; R:B_87.5:12.5_+FR, R:B_75:25_+FR, and R:B_60:40_+FR). White light with and without FR (W and W+FR) were used as control treatments comprising of 72.5% red, 19% green, and 8.5% blue light. Decreasing the R:B ratio from R:B_87.5:12.5_ to R:B_60:40_, there was a decrease in fresh weight (20%) and carbohydrate concentration (48% reduction in both sugars and starch), whereas pigment concentrations (anthocyanins, chlorophyll, and carotenoids), phenolic compounds, and various minerals all increased. These results contrasted the effects of FR supplementation in the growth spectra; when supplementing FR to different R:B backgrounds, we found a significant increase in plant fresh weight, dry weight, total soluble sugars, and starch. Additionally, FR decreased concentrations of anthocyanins, phenolic compounds, and various minerals. Although blue light and FR effects appear to directly contrast, blue and FR light did not have interactive effects together when considering plant growth, morphology, and nutritional content. Therefore, the individual benefits of increased blue light fraction and supplementary FR radiation can be combined and used cooperatively to produce crops of desired quality: adding FR increases growth and carbohydrate concentration while increasing the blue fraction increases nutritional value.

## 1 Introduction

Vertical farming (VF) is a method of controlled environmental agriculture (CEA) wherein plant production occurs in stacked layers in an enclosed growth area, without impact from the external environment (Kozai et al., 2020). In CEA, among the most controlled conditions are air temperature, nutrient solution composition, carbon dioxide concentration, and light quality (*i.e.*, spectra/wavelength of light) and quantity (*i.e.* intensity/amount of light) (SharathKumar et al., 2020; van Delden et al., 2021). As the predominant contributor to CEA start-up and production costs, light is consistent target of growers to design growth recipes minimizing production costs while maintaining or increasing yield and quality. This is now more accessible by transitioning to LEDs (light-emitting diodes) from other lighting methods (*e.g.*, high-pressure sodium lamps, fluorescent lights). The adoption of LEDs in CEA is attributed to their efficiency, reduced heat output, and production of different light wavelengths including blue light (B; 400-500 nm), red light (R; 600-700 nm), and far-red light (FR; 700-800 nm). In CEA systems with programmable and customizable LED modules (Neo et al., 2022), a plethora of custom growth recipes with specific light wavelengths, intensities, day lengths, and combinations thereof can be designed, creating an unprecedented capacity for controlling crop cultivation.

As the most effectively absorbed wavelengths by photosynthetic machinery, R and B are commonly used in VF systems. Red light is highly efficient in driving photosynthesis (McCree, 1971) and is responded to by phytochromes, photoreceptors that influence plant morphology through photomorphogenesis (Sharrock, 2008). Although R light is more cost- and energy-efficient to produce than B light, B is often needed. The B photoreceptors cryptochrome and phototropin steer plant growth by suppressing leaf expansion and stem elongation, regulating photomorphogenesis, and inducing pigment formation (Lin et al., 1998; Inoue et al., 2008; Wollaeger and Runkle, 2015). Far-red light positively increases tissue expansion and elongation, which contribute to shade avoidance (SA) mechanisms in nature (Smith and Whitelam, 1997; Keller et al., 2011). Far-red light converts phytochrome from its active FR-absorbing form (Pfr) to an inactive R-absorbing (Pr) form (Ballaré, 1999; Sharrock, 2008). Greater FR leads to a lower red:far-red ratio (R:FR), which boosts stem length, petiole length, and biomass (de Wit et al., 2016; Jin et al., 2021). In lettuce, leaf area often increases with decreasing R:FR, promoting light capture and consequent biomass (Liu and van Iersel, 2022).

Other than yield, nutritional compound concentrations also change in response to different light conditions. For example, high B exposure can increase anthocyanin concentration in some plants (Samuolienė et al., 2012; Liu et al., 2022). Anthocyanins are red- or purple-colored pigments with antioxidant activity (Khoo et al., 2017). Anthocyanins are a subclass of flavonoids (Falcone Ferreyra et al., 2012), which themselves are a subclass of phenolic compounds (Cheynier et al., 2013). Because of their antioxidant properties, phenolic compounds, flavonoids, and anthocyanins are highly sought-after health compounds in consumer foods (Sarkar and Shetty, 2014; Panche et al., 2016). In plants, they scavenge free radicals (Gould, 2004; Khoo et al., 2017), protect from ultraviolet light (UV; Woodall and Stewart, 1998), and defend from abiotic stressors (Kovinich et al., 2014, Kovinich et al., 2015; Naing and Kim, 2021).

A different class of pigments, the carotenoids, also increase with high B exposure in some plant genotypes (Samuolienė et al., 2017). Carotenoids are a group of lipid-soluble yellow/orange pigments that harvest and subsequently transfer light energy to chlorophyll for photosynthesis, also protecting chlorophyll by absorbing reactive oxygen species (ROS) (Maoka, 2020; Zulfiqar et al., 2021). In animals, carotenoids are not synthesized *de novo*, but dietary carotenoids are provitamins converted to vitamin A in the intestinal tract (Zia-Ul-Haq et al., 2021). Carbohydrates are another nutritional and energy source for plants and humans (Apriyanto et al., 2022). They can also be enriched by increasing supplemental FR in an R:B background (Van De Velde et al., 2023) or with high light intensity at the end of production (Min et al., 2021). They also affect consumer perception by increasing lettuce shelf life, sweetness, and crispness (Witkowska and Woltering, 2010; Lin et al., 2013; Min et al., 2021).

Elevating yield and enriching nutritional content via improved cultivation methods can improve antioxidant and nutrient intake of human diets (Mou, 2009). However, the solution is not simply to have high fractions of certain wavelengths; in fact, each described wavelength also has negative repercussions. High amounts of blue light (>25% B) causes dwarfed plants with reduced weight (Lin et al., 2013; Pennisi et al., 2019a; Kong and Nemali, 2021). Physiologically, FR decreases leaf thickness (Smith and Whitelam, 1997; Meng and Runkle, 2019) and FR-induced stem extension can be undesirable in crops such as lettuce as it limits leafy growth (Kong and Nemali, 2021). Metabolically, FR irradiance has been shown to decrease phenolic, anthocyanin, carotenoid, and chlorophyll content (Li and Kubota, 2009; Meng and Runkle, 2019). Finally, crops exposed solely to R exhibit a “red light syndrome”, characterized by hampered photosynthesis, biomass accumulation, and morphology (Hogewoning et al., 2010; Trouwborst et al., 2016). This response is mitigated by adding small B fractions, restoring normal photosynthetic and growth functionality (Hoenecke et al., 1992; Hogewoning et al., 2010). Furthermore, the interactions between R/B/FR wavelengths on plant physiology and nutrition are less clear than when focused on individual wavelengths. Blue-sensing cryptochromes and R/FR-sensing phytochromes have complex interactions in light response; in some conditions working cooperatively (Su et al., 2017), in others antagonistically (Mockler et al., 1999), and still other instances acting independently (Hirose et al., 2012; Casal, 2013).

The mixed bag of plant responses to different treatments of R, B, and FR light exemplifies the need to fine-tune crop production through custom LED light recipes focused on growth and desired compound biosynthesis. However, to our knowledge, the growth and nutritional effects of R:B ratios interacting with a constant intensity of supplemental FR has yet to be described. Previous studies comparing lettuce growth and pigments under different R:B and FR conditions have either primarily focused on very young lettuce plants (Meng and Runkle, 2019) or have compared R:B:FR conditions with different total R and B content depending on the inclusion or exclusion of FR (Kong and Nemali, 2021). Because the application of FR with R:B has not thus far been performed consistently, it is unclear if a constant intensity of supplemental FR combined with different R:B ratios may interact cooperatively, negatively, or independently. Therefore, we sought to fill this knowledge gap by applying a constant intensity of supplemental FR with a range of R:B ratios, focusing on growth and nutritional content of lettuce at a harvestable and nutritionally-relevant developmental stage.

In this study, we were interested in the effect of R:B and FR on economically attractive traits, with focus on identifying “balanced” conditions where nutritional value could be promoted whilst maintaining suitable plant growth. Our objective was to quantify the yield and nutritional value of red lettuce grown under LEDs at different R:B ratios with or without FR, additionally determining if the R:B ratios had interactions with FR. To perform this, we applied four treatments with increasing B content in the R:B spectrum, then included or excluded supplemental FR light. Three of these spectra used only R and B light to create the treatment R:B spectra. The fourth treatment spectra was a white light treatment with a high R:B ratio, which was used as a reference spectra with a known high R and low B content, as performed in previous studies (Pennisi et al., 2019b; Ji et al., 2021). Here, pigments (anthocyanins, chlorophyll, and carotenoids), phenolic compounds, carbohydrates, and mineral concentration were quantified as markers for lettuce nutritional value. We hypothesized that FR addition to R:B growth spectra would improve biomass accumulation, albeit with reductions in nutritional value corresponding to decreased foliage pigmentation and phenolic content. The opposite was hypothesized for increasing B fractions, which were conversely expected to decrease biomass accumulation while improving nutritional content. Additionally, we intended to determine if physiological and nutritional phenotypes were results of cooperative, antagonistic, or discrete light responses to B and FR.

## 2 Materials and methods

### 2.1 Plant material and germination

*Lactuca sativa* cv. Barlach (Rijk Zwaan; De Lier, The Netherlands), a red butterhead lettuce, was grown from pelleted seeds sown individually on rockwool plugs (3.5 × 3.5 × 5.9 cm L × W × H; Grodan, Roermond, The Netherlands), covered with a layer of vermiculite. The plugs were imbibed with tap water and kept in a germination tray covered with clear plastic to maintain humidity. The germination trays were placed in darkness for two days at 4°C for stratification, then were moved to a climate room equipped with a CO_2_ supplier (800 ppm CO_2_) and an air-conditioning system for controlling room temperature (21°C /19°C day/night) and relative humidity (75%). Plants were germinated under white light (GreenPower LED production module deep red/white 150, 2^nd^ generation; Philips, Eindhoven, The Netherlands) for five days at 200 µmol m^-2^ s^-1^ PAR, 18 h light/6h dark. The total incident light intensity (µmol m^-2^ s^-1^ PAR) was measured at plant height using a PAR meter (LI-250A; Li-Cor Biosciences, Lincoln, NE, USA). The spectral photon composition was 8.5% blue (400-500 nm), 19% green-yellow (500-600 nm), 72.5% red (600-700 nm), and 0% FR (700-800 nm) during germination. Spectral composition was measured with a spectrometer (SS-110; Apogee Instruments, Logan, UT, USA).

### 2.2 Growth conditions

Seven days after sowing, rockwool plugs with morphologically-similar seedlings and two unfurled cotyledons were transplanted to water-soaked rockwool blocks (7.5 × 7.5 × 6.5 cm, L × W × H; Grodan, Roermond, The Netherlands). Eight groups of 22 plants were arranged in individual growth compartments (0.82 m^2^, 27 plants m^-2^) with different light treatments (Section 3.3), and plant positions were randomized weekly within a compartment. All plants continued to be grown at 21°C /19°C (day/night), 75% relative humidity, and 800 ppm CO_2_. Every three days, plants received a nutrient solution (as used in Jin et al., 2021) containing 12.92 mM NO_3_^−^, 8.82 mM K^+^, 4.22 mM Ca^2+^, 1.53 mM Cl^−^, 1.53 mM SO_4_^2−^, 1.53 mM H_2_PO_4_^−^, 1.15 mM Mg^2+^, 0.38 mM NH_4_^+^, 0.38 mM SiO_3_^2−^, 0.12 mM HCO_3_^−^, 38.33 μM B, 30.67 μM Fe_3_^+^, 3.83 μM Mn_2_^+^, 3.83 μM Zn^2+^, 0.77 μM Cu^2+^, and 0.38 μM Mo, at an EC of 2.3 dS m^-1^ and pH 6-6.5. As this solution has a greater osmotic pressure than water, seedlings were adapted to it by diluting the nutrient solution with water to EC values of 0.5, 1.0, 1.5, and 2.0 at 0, 2, 4, and 6 days after transplanting, respectively. Nutrient solution EC was verified using an EC meter (Elmeco EC handmeter V2.0, Tasseron, Nootdorp, The Netherlands).

### 2.3 Light treatments

Plants were grown at 200 µmol m^-2^ s^-1^ PAR (18 h light/6h dark) under either white light (W) or one of three R:B ratios: 87.5:12.5 (R:B_87.5:12.5_, 12.5% B), 75:25 (R:B_75:25_, 25% B), or 60:40 (R:B_60:40_, 40% B), either with or without far-red addition (+FR; 50 µmol m^-2^ s^-1^; separate from, but equivalent to 25% PAR) (**Table 1**; **Supplementary Figure 1**). PAR was provided by combinations of Greenpower LEDs (GreenPower LED production module deep red/white 150, GP LED production DR/B 150 LB, GP LED production B 120 LO, 1^st^ and 2^nd^ generation; Philips, Eindhoven, The Netherlands); FR was provided by GreenPower LED production module far red 150cm (Philips, Eindhoven, The Netherlands). PAR was maintained at 200 µmol m^-2^ s^-1^ by adjusting the height of the LED modules suspended above the plant canopy. For brevity, when presenting and discussing results, the treatments (R:B_87.5:12.5_, R:B_75:25_, R:B_60:40_) and the resulting data are referred to by the blue content in the R:B spectrum; importantly, this means in this context that increased B content also corresponds to reduced R content. This is not exactly the same for W treatments, which also involve green-yellow wavelengths. However, W treatments were analyzed as a good comparison treatment to consider the effect of a lower content of B and R with the presence of green-yellow. Therefore, W treatments here were used as a reference treatment, an approach performed in other previous light studies (Pennisi et al., 2019b; Ji et al., 2021).

**Table 1:** Spectral compositions, light intensity, and phytochrome photostationary state of eight different light treatments.

Treatment	Spectral composition of PAR (%)			Intensity (µmol m^-2^ s^-1^)		R:B	R:FR	PSS value
	Blue ^400-500 nm^	Green-Yellow ^500-600 nm^	Red ^600-700 nm^	PAR ^400-700 nm^	Far-red ^700-800 nm^			
Germination	8.5	19.0	72.5	200	0	8.53	N/A	0.88
W	8.5	19.0	72.5	200	0	8.53	N/A	0.88
R:B_87.5:12.5_	12.5	0.0	87.5	200	0	7.00	N/A	0.88
R:B_75:25_	25.0	0.0	75.0	200	0	3.00	N/A	0.88
R:B_60:40_	40.0	0.0	60.0	200	0	1.50	N/A	0.87
W+FR	8.5	19.0	72.5	200	50	8.53	1.45	0.81
R:B_87.5:12.5_+FR	12.5	0.0	87.5	200	50	7.00	1.75	0.82
R:B_75:25_+FR	25.0	0.0	75.0	200	50	3.00	1.50	0.81
R:B_60:40_+FR	40.0	0.0	60.0	200	50	1.50	1.20	0.78

W, white light; R:B_87.5:12.5_, R:B_75:25_, R:B_60:40_, R:B ratios used in this study; FR, supplemental far-red light; PAR, photosynthetically active radiation; R:FR, red:far-red ratio; PSS, phytochrome photostationary state, calculated using the different spectral treatments at 200 µmol m^-2^ s^-1^ PAR.

N/A, not applicable.

### 2.4 Morphological measurement

Non-destructive morphological measurements were performed 7, 14, and 21 days after transplant (DAT) for five growth cycles. These measurements consisted of projected leaf area (PLA; the area of leaves exposed to light in cm^2^ plant^-1^) and number of leaves (L_N_; # plant^-1^). For PLA measurements (and morphological characteristics), overhead photos of individual plants were taken using a stand-mounted digital camera (EOS 1100D, Canon, Tokyo, Japan). Photos were captured with a black background and size reference, with which PLA was calculated using ImageJ (U. S. National Institutes of Health, Bethesda, MD, USA). Leaf number was determined by counting all leaves >0.5 cm^2^.

For further morphological and metabolic data collection, plants from three growth cycles were harvested 21 DAT. Of the 22 plants per treatment per growth cycle, ten plants were randomly selected for morphological measurements: leaf area (cm^2^ plant^-1^), shoot fresh weight (g plant^-1^), and shoot dry weight (g plant^-1^). Shoot fresh weight and leaf area was determined by separating leaves and measuring with a leaf area meter (LI-3100, Li-Cor Biosciences, Lincoln, NE, USA). These same leaves and stems were used for shoot dry weight determination after being dehydrated in a forced-air oven at 105°C for 48 hours (Elbanton Special Products by Hettich Benelux, Geldermalsen, The Netherlands). With these data, specific leaf area (SLA; cm^2^ g^-1^) and dry matter content (%) were calculated. From the remaining twelve plants per treatment and growth cycle, eight plants were used for anthocyanin determination and four were used for other metabolite analysis (Section 3.5).

### 2.5 Metabolic compound measurements

From light-exposed regions of leaves, four leaf disks (~1cm diameter) from each plant were collected and weighed in Eppendorf tubes containing ceramic grinding balls, then flash frozen in liquid nitrogen and stored at -80°C. Two tubes were created per treatment, one for anthocyanin analysis and one for other metabolite analysis. After freezing, leaf disks were subsequently freeze-dried (Alpha 1-4 LSCbasic, Martin Christ, Osterode am Harz, Germany) for a minimum of 36 hours, then ground into a fine powder with the preloaded balls using a mixer mill (MM 200, Retsch, Dale i Sunnfjord, Norway). The leaf disk fresh weights were measured prior to freezing, therefore the resulting data for metabolite concentrations are expressed per gram of fresh weight.

Anthocyanins were measured following previous descriptions, with some modifications (Lange et al., 1971; Wu et al., 2014). Anthocyanins were extracted from ~10 mg of freeze-dried and ground leaf material using 1 mL of extraction buffer (18% [v/v] 1-propanol, 1% [v/v] HCl and 81% [v/v] MilliQ water). Samples and blanks (no plant tissue, only buffer) were boiled for three minutes at 100°C and incubated for two hours in the dark at room temperature, then centrifuged. Sample absorbances at 535 nm (A_535_) and 650 nm (A_650_) were determined using a spectrometer (SpectraMax iD5, San Jose, CA, USA) and corrected with blank values. Anthocyanin content was measured as (A_535_-2.2·A_650_)/mg FW.

Chlorophyll a (Chl_a_), chlorophyll b (Chl_b_), total chlorophyll (Chl_a+b_), malondialdehyde (MDA), total phenolic compounds, total flavonoids, sugars, and starch concentrations were measured according to López-Hidalgo et al. (2021), using ~10 mg of freeze-dried tissue.

### 2.6 Mineral concentration measurement

For mineral composition analysis, plants from two growth cycles were harvested 21 days after transplantation. To achieve the necessary 200 grams of cumulative fresh shoot biomass for mineral analysis, multiple plants were collected for each treatment, noting the number and fresh weight of individual plants required to satisfy 200 grams of tissue (10 to 26 plants, depending on the plant fresh weight from different treatments). Mineral composition was determined by a certified nutrient testing company (Eurofins Agro NL, Wageningen, The Netherlands), determining macro- and micro-element concentrations for plants in each treatment.

### 2.7 Radiation- and energy-use efficiency calculations

Radiation-use efficiency and energy-use efficiency were calculated based on typical performance values of photon efficacy for LED packages (B = 2.8 µmol J^-1^, R = 4.1 µmol J^-1^, FR = 3.6 µmol J^-1^, white = 2.8 µmol J^-1^) (Kusuma et al., 2022). These were calculated considering plant fresh weight by treatment, plant density (27 plants m^-2^), treatment duration (21 days), and total radiation received within the 400-800 nm range.

### 2.8 Experimental design and statistical analyses

The experiment was conducted five times in a row, resulting in five growth cycles. Three growth cycles were used for morphological and metabolic data collection, therefore for these measurements there were three statistical replicates (*n* = 3). Two growth cycles were used for mineral data collection, therefore for these measurements there were two statistical replicates (*n* = 2). The growth cycles were treated as blocks in the statistical analysis; each light treatment was randomly allocated to a growth compartment, but due to technical restrictions, the same randomization was used in each growth cycle. For each growth cycle, from the 22 plants grown under each treatment, ten plants were used for collecting morphological data, eight plants for anthocyanin analysis, and four plants for metabolite determination as in López-Hidalgo et al. (2021). For both mineral content growth cycles, one measurement per aggregate set of plants from each light treatment was made. For every variate, a two-way analysis of variance (ANOVA) was conducted (factors FR and background light spectrum, each with two levels), taking account for the blocks was conducted. This was followed by mean separation according to Fisher’s unprotected least significant difference (LSD) test. Furthermore, a two-way ANOVA in blocks was conducted using FR presence as a qualitative factor and B% in the spectrum (excluding the white light treatments) as a quantitative factor with polynomial contrasts. All tests were conducted at α *=* 0.05 and Genstat software (21^st^ edition, VSN International LTD, Hemel Hempstead, UK) was used for all statistical tests.

## 3 Results

### 3.1 Supplemental FR improves plant growth in R:B backgrounds

**Figure 1:**
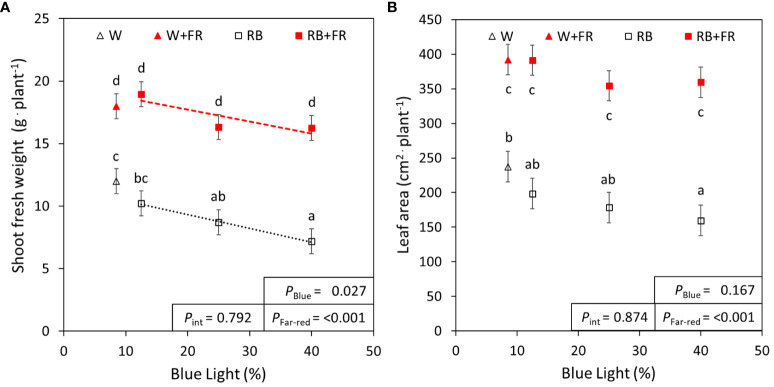
Lettuce fresh weight and leaf area under red:blue ratios with and without far-red. **(A)** Shoot fresh weight and **(B)** leaf area of lettuce grown under three different red:blue ratios presented by blue light % in the R:B spectrum (R:B_87.5:12.5_ = 12.5% blue; R:B_75:25_ = 25% blue; and R:B_60:40_ = 40% blue), with additional far-red light (RB+FR) or no far-red (RB). White light (~8.5% blue) is used as a comparison, with far-red (W+FR) or without (W). Trendlines were drawn to indicate the probability of a linear relationship with blue light (*P*_Blue_, α = 0.05). Different letters indicate significantly different values for each combination of R:B ratio and FR light treatments, according to an unprotected Fisher LSD Test (α = 0.05). Datapoints represent treatment means with error bars representing standard error means of three growth cycles (*n* = 3), each consisting of ten replicate plants. *P*_Far-red_ = probability of an effect from far-red, *P*_int_ = probability of an interactive effect between blue content and far-red addition.

As important parameters for crop production, the morphological traits of lettuce grown under different R:B and FR treatments were measured. Considering the different R:B ratios, shoot fresh weight decreased by 20% from R:B_87.5:12.5_ to R:B_60:40_ treatments, at similar magnitudes with or without FR (**Figure 1A**). With the addition of 50 µmol m^-2^ s^-1^ FR radiation, shoot fresh weight increased by 100% on average for each R:B ratio. There was no statistical interaction found between FR addition and B content on shoot fresh weight. There was also no effect on dry matter content for both B content and FR addition, resulting in similar trends observed between shoot fresh and dry weight (**Table 2**; **Supplementary Figure 2A**). Adding FR to R:B treatments resulted in a 106% increase of total leaf area over treatments without FR (**Figure 1B**). There did appear to be a trend of decreased leaf area with increased B content, but this was not found to be significant (**Figure 1B**). Specific leaf area increased linearly with higher blue light percentage (**Table 2**); as SLA is inversely proportional to leaf thickness, leaves were thinner as B content increased, made slightly thinner when FR was added (**Table 2**). Canopy openness (total projected leaf area divided by leaf area) slightly decreased with supplemental FR but was unaffected by B content (**Table 2**). The number of leaves per plant increased with supplemental FR and did not appear to be affected by B content, except for the R:B_75:25_+FR treatment, which had a lower number of leaves compared to R:B_87.5:12.5_+FR and R:B_60:40_+FR (**Table 2**).

**Table 2:** Morphological characteristics of lettuce grown under different light spectra.

	Unit	FR	W	R:B_87.5:12.5_	R:B_75:25_	R:B_60:40_	SEM^ǂ^	*P*_Blue_^#^	*P*_Far-red_^##^
DMC	%	No FR	4.95^a^	5.09^a^	5.22^a^	5.12^a^	± 0.153	0.649	0.081
		+FR	4.98^a^	4.88^a^	5.00^a^	4.97^a^			
PLA	cm^2^ plant^-1^	No FR	50.63^a^	56.26^a^	61.55^ab^	73.84^b^	± 5.13	0.220	<0.001^*^
		+FR	116.49^c^	112.84^c^	108.41^c^	106.03^c^			
$\frac{\text{PLA}}{\text{LA}}$		No FR	0.350^ab^	0.356^ab^	0.349^ab^	0.365^b^	± 0.011	0.942	0.030^*^
		+FR	0.336^ab^	0.336^ab^	0.339^ab^	0.325^a^			
SLA	cm^2^ g^-1^	No FR	404.0^abc^	386.0^a^	401.2^ab^	444.4^bc^	± 16.36	0.010^*^	0.022^*^
		+FR	444.9^bc^	427.2^abc^	439.5^bc^	451.0^c^			
No. of leaves	# plant^-1^	No FR	15.89^a^	15.39^a^	14.46^a^	15.19^a^	± 0.587	0.895	<0.001^*^
		+FR	19.80^bc^	20.10^bc^	18.39^b^	20.15^c^			

DMC, shoot dry matter content; PLA, projected leaf area; PLA/LA, leaf canopy closure; SLA, specific leaf area; W, white light; R:B_87.5:12.5_, R:B_75:25_, R:B_60:40_, R:B ratios used in this study; FR, supplemental far-red light.

**^ǂ^** SEM, standard error means of three growth cycles (n = 3), each consisting of ten replicate plants for all eight light treatments. Different letters indicate significantly different values for each combination of R:B ratio and FR light treatments, using an unprotected Fisher LSD Test (α = 0.05).

**^#^** P-value for blue content effects among the three levels of blue light according to a two-way ANOVA.

**^##^** P-value for far-red light effects among the three levels of blue light according to a two-way ANOVA.

*Denotes a significant effect of either P^Blue^ or P^Far-red^ (α = 0.05).

### 3.2 Leaf pigments increase with increased B, but not FR, in an R:B background

**Figure 2:**
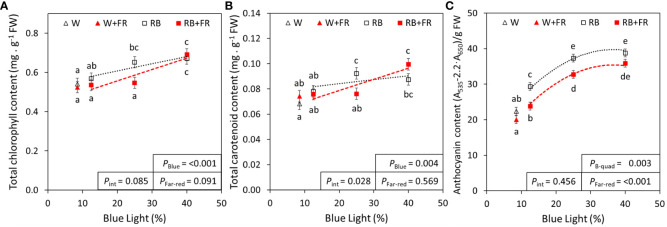
Pigment concentrations of lettuce grown under red:blue treatments with and without far-red. Total chlorophyll **(A)**, carotenoid **(B)**, and anthocyanin **(C)** concentrations of lettuce grown under different red:blue ratios presented by blue light % in the R:B spectrum (R:B_87.5:12.5_ = 12.5% blue; R:B_75:25_ = 25% blue; and R:B_60:40_ = 40% blue), with additional far-red light (RB+FR) or no far-red (RB). White light (~8.5% blue) is used as a comparison, with far-red (W+FR) or without (W). Trendlines were drawn to indicate the probability of a linear (*P*_Blue_) or quadratic (*P*_B-quad_) relationship with blue light, α = 0.05. Different letters indicate significantly different values for each combination of R:B ratio and FR light treatments, according to an unprotected Fisher LSD Test (α = 0.05). Datapoints represent treatment means with error bars representing standard error means of three growth cycles (*n* = 3), each consisting of four or eight replicate plants. *P*_Far-red_ = probability of an effect from far-red, *P*_int_ = probability of an interactive effect between blue content and far-red addition.

**Figure 3:**
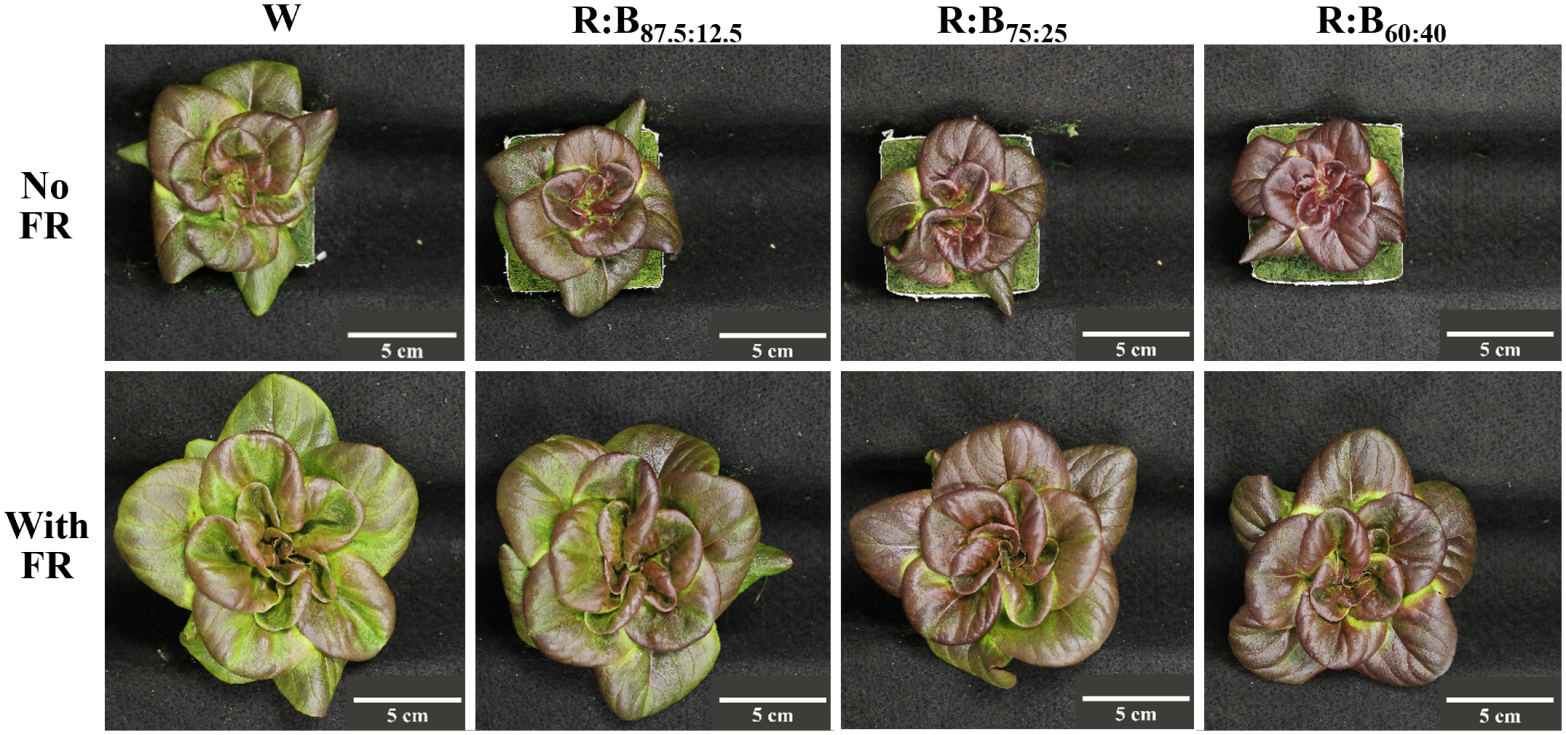
Morphology of “Barlach” lettuce grown under different light conditions. Left to right: Representative photographs of lettuce grown under white light (W) or three R:B light ratios (R:B_87.5:12.5_, R:B_75:25_, and R:B_60:40_). The percentage of blue in PAR for each treatment was 8.5%, 12.5%, 25%, and 40%, respectively. Plants were grown without far-red (top row, No FR) and with 50 µmol m^-2^ s^-1^ supplementary far-red (bottom row, With FR).

To ascertain the differences in lettuce pigmentation between treatments, photosynthetic and photoprotective pigment contents were quantified. Generally, leaf pigments increased with increased B content in an R:B spectrum (**Figure 2**). From R:B_87.5:12.5_ to R:B_60:40_, there was linear increase of chlorophyll (24%) and carotenoid (21%) concentration; these pigments were not significantly affected by adding FR (**Figures 2A, B**). However, there was an interaction when adding FR to an R:B background for carotenoid concentration. The Chl_a_:Chl_b_ ratio decreased slightly from R:B_87.5:12.5_ to R:B_60:40_, further decreased with addition of FR (**Supplementary Table 1**). Anthocyanins increased by 40% from R:B_87.5:12.5_ to R:B_60:40_ independently of FR, which decreased anthocyanin content for all R:B treatments by 13% (**Figure 2C**). The effects of FR and B content on anthocyanins can also be visualized in **Figure 3**, as the red color of red lettuce can be a proxy for relative anthocyanin content.

### 3.3 Specialized metabolites, carbohydrates, and minerals are differentially affected by R:B ratios and FR supplementation

**Figure 4:**
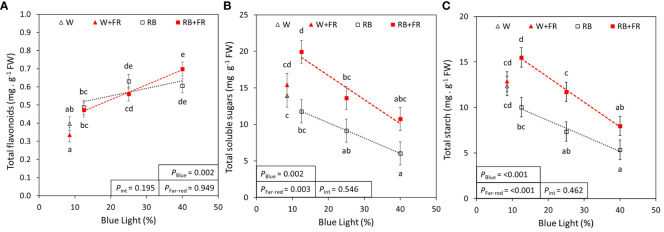
Blue content and far-red effects on total flavonoids, soluble sugars, and starch. Total flavonoid **(A)**, soluble sugar **(B)**, and starch **(C)** concentrations of lettuce grown under grown under different red:blue ratios presented by blue light % in the R:B spectrum (R:B_87.5:12.5_ = 12.5% blue; R:B_75:25_ = 25% blue; and R:B_60:40_ = 40% blue), with additional far-red light (RB+FR) or no far-red (RB). White light (~8.5% blue) is used as a comparison, with far-red (W+FR) or without (W). Trendlines were drawn to indicate the probability of a linear relationship with blue light (*P*_Blue_, α = 0.05). Different letters indicate significantly different values for each combination of R:B ratio and FR light treatments, according to an unprotected Fisher LSD Test (α = 0.05). Datapoints represent treatment means with error bars representing standard error means of three growth cycles (*n* = 3), each consisting of four replicate plants. *P*_Far-red_ = probability of an effect from far-red, *P*_int_ = probability of an interactive effect between blue content and far-red addition.

To quantify additional indicators of nutritional value, the concentrations of flavonoids, phenolic compounds, carbohydrates, and minerals were assessed. The concentrations of total flavonoids and phenolic compounds increased linearly from R:B_87.5:12.5_ to R:B_60:40_ by 35% and 20%, respectively; however, neither were significantly affected with supplemental FR (**Figure 4A**; **Supplementary Figure 2B**). There appeared to be no effect of FR or B content on MDA concentration (**Supplementary Figure 2C**). For carbohydrates, total soluble sugars and starch showed strong linear decreases from R:B_87.5:12.5_ to R:B_60:40_ (both 47%, **Figures 4B, C**). On average, with supplemental FR, soluble sugars increased by 65% and starch increased by 54% over treatments without FR. For each of flavonoids, phenolic compounds, MDA, sugars, and starch, no interaction was found between blue light percentage and FR.

**Figure 5:**
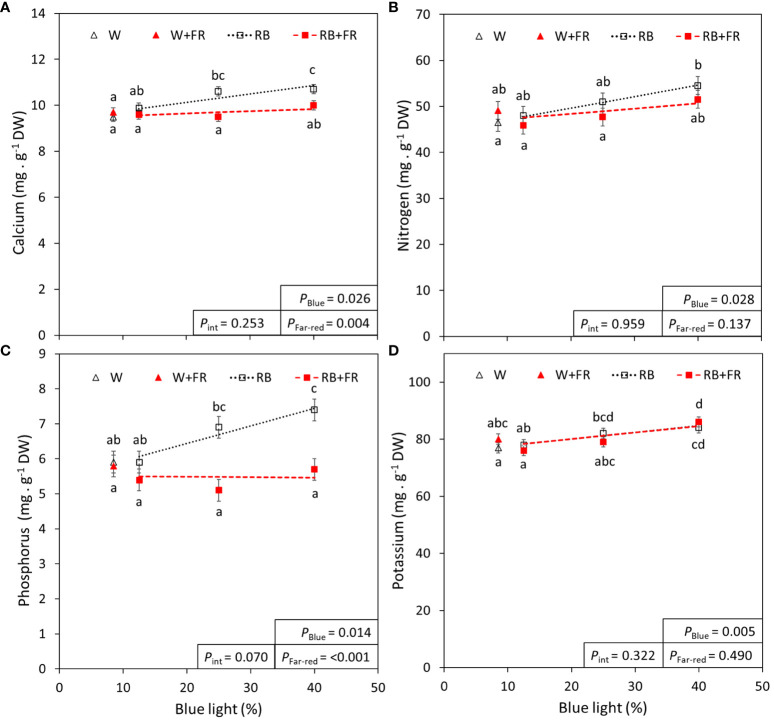
Blue light and far-red light effects on calcium, nitrogen, phosphorus, and potassium. The concentration of calcium **(A)**, nitrogen **(B)**, phosphorus **(C)**, and potassium **(D)** in lettuce grown under different red:blue ratios presented by blue light % in the R:B spectrum (R:B_87.5:12.5_ = 12.5% blue; R:B_75:25_ = 25% blue; and R:B_60:40_ = 40% blue), with additional far-red light (RB+FR) or no far-red (RB). White light (~8.5% blue) is used as a comparison, with far-red (W+FR) or without (W). Trendlines were drawn to indicate the probability of a linear relationship with blue light (*P*_Blue_, α = 0.05). Different letters indicate significantly different values for each combination of R:B ratio and FR light treatments, according to an unprotected Fisher LSD Test (α = 0.05). Datapoints represent treatment means with error bars representing standard error means of two growth cycles (*n* = 2) with measurements corresponding to an aggregate set of plants from each treatment. *P*_Far-red_ = probability of an effect from far-red, *P*_int_ = probability of an interactive effect between blue content and far-red addition.

Of the analyzed macro- and micro-elements, four are showcased in **Figure 5** due to their use in fertilizers (nitrogen, phosphorus, and potassium) or involvement in lettuce tipburn studies (calcium) (Frantz et al., 2004). The remainder of the analyzed elements are summarized in **Table 3**. Most macro- and micro-elements increased linearly with increased B content, except for magnesium, chloride, iron, and boron. With added FR, there were significant decreases in the mineral concentrations of calcium, magnesium, manganese, and phosphorus. Generally, the increase in mineral concentration due to increased B content was often greater than the decrease in mineral concentration due to FR addition. Finally, when considering the whole plant nutrient content (which was calculated from the measured concentrations in **Table 3**), there was a significant effect of FR for each mineral (**Supplementary Table 2**).

**Table 3:** Macro- and micro-elements in lettuce grown under different light spectra.

Element	FR	W	R:B ^87.5:12.5^	R:B ^75:25^	R:B ^60:40^	SEM^ǂ^	*P*_Blue_^#^	*P*_Far-red_^##^	*P*_int_^###^
Boron (µg g^-1^ DW)	No FR	23.2^a^	23.1^a^	23.2^a^	23.9^a^	± 0.64	0.076	1.000	0.683
	+FR	23.3^a^	22.4^a^	23.4^a^	24.2^a^				
Chloride (mg g^-1^ DW)	No FR	7.0^a^	7.3^a^	7.4^a^	7.2^a^	± 0.19	0.471	1.000	0.840
	+FR	7.3^a^	7.3^a^	7.5^a^	7.2^a^				
Copper (µg g^-1^ DW)	No FR	7.5^a^	7.6^a^	9.1^c^	9.6^c^	± 0.36	0.005^*^	0.060	0.608
	+FR	7.6^a^	7.1^a^	7.8^ab^	9.0^bc^				
Iron (µg g^-1^ DW)	No FR	160^a^	145^a^	260^b^	195^ab^	± 29.4	0.392	0.475	0.374
	+FR	135^a^	165^ab^	185^ab^	190^ab^				
Magnesium (mg g^-1^ DW)	No FR	2.3^a^	2.5^ab^	2.7^b^	2.8^b^	± 0.09	0.143	0.011^*^	0.606
	+FR	2.4^a^	2.4^a^	2.4^a^	2.5^a^				
Manganese (µg g^-1^ DW)	No FR	36^ab^	34^ab^	40^bc^	42^c^	± 2.0	0.021^*^	0.003^*^	0.223
	+FR	32^a^	30^a^	30^a^	32^a^				
Molybdenum (µg g^-1^ DW)	No FR	0.8^a^	0.8^a^	0.9^a^	1.0^ab^	± 0.09	0.025^*^	0.518	0.218
	+FR	0.9^a^	0.8^a^	0.8^a^	1.2^b^				
Nitrate (mg g^-1^ DW)	No FR	30.6^a^	30.8^a^	34.7^ab^	41.5^ab^	± 3.73	0.027^*^	0.821	0.737
	+FR	37.7^ab^	32.2^a^	32.0^a^	44.9^b^				
Sodium (mg g^-1^ DW)	No FR	0.7^a^	0.7^a^	0.9^ab^	1.0^b^	± 0.05	0.013^*^	0.465	0.572
	+FR	0.8^a^	0.7^a^	0.9^ab^	0.9^ab^				
Sulfur (mg g^-1^ DW)	No FR	2.7^a^	2.8^ab^	3.0^bc^	3.2^c^	± 0.06	<0.001^*^	0.156	0.508
	+FR	2.9^ab^	2.8^ab^	2.9^ab^	3.2^c^				
Zinc (µg g^-1^ DW)	No FR	24^ab^	22^a^	26^abc^	28^bc^	± 1.5	0.013^*^	0.578	0.542
	+FR	24^ab^	24^ab^	25^ab^	30^c^				

W, white light; R:B_87.5:12.5_, R:B_75:25_, R:B_60:40_, R:B ratios used in this study; FR, supplemental far-red light; DW, dry weight.

**^ǂ^** SEM, standard error means of two growth cycles (n = 2), consisting of multiple plants (10 to 26, depending on treatment) for all eight light treatments. Different letters indicate significantly different values for each combination of R:B ratio and FR light treatments, using an unprotected Fisher LSD Test (α = 0.05).

**^#^** P-value for blue content effects among the three levels of blue light according to a two-way ANOVA.

**^##^** P-value for far-red light effects among the three levels of blue light according to a two-way ANOVA.

**^###^** P-value for interactive effects between far-red and blue light according to a two-way ANOVA.

^*^Denotes a significant effect of either P_Blue_ or P_Far-red_ (α = 0.05).

### 3.4 White light treatments were not significantly different from blue light trends in R:B spectra

Overall, for most analyzed parameters, the effects of white light (W and W+FR) were not significantly different from the closest B light content R:B ratio (R:B_87.5:12.5_ and R:B_87.5:12.5_+FR), except for anthocyanin (**Figure 2C**) and flavonoid (**Figure 4A**) concentration. Anthocyanin content was lower in W compared to R:B_87.5:12.5_ and W+FR was lower than R:B_87.5:12.5_+FR. For flavonoids, only W+FR was significantly lower than R:B_87.5:12.5_+FR. For all other parameters, W followed R:B considering its B light content (~8.5%) and W+FR followed R:B+FR.

### 3.5 Energy-use efficiency of R:B ratios with and without additional FR

**Figure 6:**
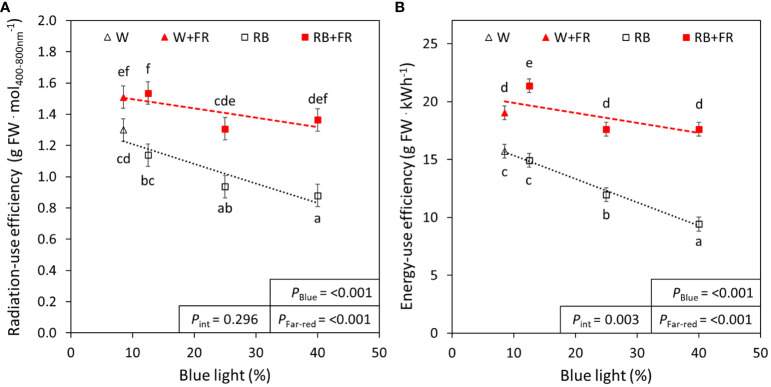
Radiation-use efficiency and energy-use efficiency of red:blue and far-red treatments. The radiation-use efficiency **(A)** and energy-use efficiency **(B)** of different red:blue ratios presented by blue light % in the R:B spectrum (R:B_87.5:12.5_ = 12.5% blue; R:B_75:25_ = 25% blue; and R:B_60:40_ = 40% blue), with additional far-red light (RB+FR) or no far-red (RB). White light (~8.5% blue) is used as a comparison, with far-red (W+FR) or without (W). Trendlines were drawn to indicate the probability of a linear relationship with blue light (*P*_Blue_, α = 0.05). Different letters indicate significantly different values for each combination of R:B ratio and FR light treatments, according to an unprotected Fisher LSD Test (α = 0.05). Datapoints represent treatment means with error bars representing standard error means of three growth cycles (*n* = 3), each consisting of ten replicate plants. *P*_Far-red_ = probability of an effect from far-red, *P*_int_ = probability of an interactive effect between blue content and far-red addition.

As B light content increased in the treatments without FR, there was a significant decrease in radiation-use efficiency (21%) and energy-use efficiency (26%) (**Figure 6**). These decreases due to B content were not significant in treatments with additional FR (**Figure 6**). Additional FR increased both radiation-use efficiency from 17% to 59% (depending on B content) and energy-use efficiency from 20% to 87% (depending on B content) (**Figure 6**).

## 4 Discussion

### 4.1 FR and increased B content have contrasting roles in morphology

Overall, an increased B fraction in R:B decreased lettuce shoot fresh weight, dry weight, and leaf area, while adding FR to each R:B ratio consistently increased each of these parameters (**Figure 1**; **Supplementary Figure 2A**). Smaller plants due to increased B light content has been previously described (Wollaeger and Runkle, 2015; Pennisi et al., 2019b; Kong and Nemali, 2021), suggested by Snowden et al. (2016) to be due to reduced radiation capture from smaller leaves. Conversely, FR characteristically extends stems, internodes, petioles, and expands leaves, causing greater light interception, resulting in increased overall growth (Devlin et al., 1998; Keller et al., 2011; de Wit et al., 2016; Jin et al., 2021). The red-leafed lettuce in this study showed leaf expansion due to FR content, which has been shown to occur in many cultivars of lettuce and tomato, but not all (Ji et al., 2021; Liu and van Iersel, 2022). Increased leaf area (**Figure 1B**) without changing dry matter content (**Table 2**) can increase light interception, enhancing growth with FR addition (**Supplementary Figure 3**). Increased B content corresponded with decreased leaf thickness (higher SLA), contrary to commonly observed thicker leaves in many plant species (Wollaeger and Runkle, 2015; Shengxin et al., 2016; Zheng and Van Labeke, 2017). However, decreased leaf thickness with increased B has been found more often in lettuce (Clavijo-Herrera et al., 2018; Pennisi et al., 2019b; Kong and Nemali, 2021). Plants with supplemental FR also had slightly thinner—but larger—leaves (**Table 2**), which has been seen to occur with FR presence (Smith and Whitelam, 1997; Keller et al., 2011; Meng and Runkle, 2019). Interestingly, FR also increased leaf number, although literature shows that FR inclusion often decreases or does not affect leaf number, which can be species- or genotype-specific (Ji et al., 2020; Jin et al., 2021; Kong and Nemali, 2021). The combined positive effects of FR with a low R:B ratio on yield, leaf area, and morphology exceeded the negative impact of increased B in an R:B background. Therefore, FR addition can positively impact tissue production, plant size, and shoot fresh and dry weight under decreasing R:B ratios (which would otherwise negatively impact these parameters), allowing for growers to improve factors such as nutritional value with different R:B conditions.

### 4.2 Antioxidants are induced with greater B content, but differentially affected by FR addition

Apart from leaf size, the most prominent lettuce phenotype observed was a progressively deeper, redder, pigmentation with increased B content, especially in treatments without FR (**Figure 3**). The deeper red is linked to the production of anthocyanins, associated with human health because of their antioxidant capacity (Sarkar and Shetty, 2014; Panche et al., 2016; Khoo et al., 2017). In our analysis, anthocyanins increased as B content increased, which has been found to occur via upregulation from the activated cryptochrome photoreceptor CRY1 (Bouly et al., 2007). This upregulation aligns with anthocyanin function, protecting plants from reduced photosynthesis from photoinhibition, often caused by stressors such as a greater incidence of higher-intensity blue light (Smillie and Hetherington, 1999). Increased anthocyanin content with greater blue light fraction has also been found in a variety of species and organs including pepper fruit (Liu et al., 2022) strawberry fruit (Zhang et al., 2018), and tea leaves (Zheng et al., 2019). Here, anthocyanin content decreased with added FR, which has been previously described in lettuce (Kong and Nemali, 2021). However, the decrease in anthocyanin content with added FR is noticeably lower than the increase in fresh weight of FR-grown lettuce; therefore, larger plants grown under FR can still gain deeper pigmentation and nutritional benefits from low R:B ratios.

As they are also antioxidants associated with health-promoting activity (Sarkar and Shetty, 2014; Panche et al., 2016), flavonoid and phenolic compound concentrations were explored. In this study, total flavonoids and phenolic compounds were both found to increase with elevated B in an R:B background. Interestingly, although anthocyanins had a quadratic response to increased B light (**Figure 2C**), total flavonoid and phenolic compound concentrations had linear responses (**Figure 4A**; **Supplementary Figure 2B**), suggesting a point of diminishing returns for anthocyanin production serving for light protection. Although the photoprotective anthocyanins expectedly increased as high-energy B increases in R:B, at a certain point, anthocyanin content appeared to plateau, whereas flavonoids and phenolic compounds steadily increased with higher B. This may indicate that past the point of anthocyanin production plateau, the metabolism of other antioxidants and phenolic compounds may become of primary focus, to tackle existing ROS created by oxidative stress (Cheynier et al., 2013; Dumanović et al., 2021), in this case caused by the high-energy B light incidence. As MDA normally accumulates as a breakdown product of ROS-induced lipid peroxidation of hydroperoxides (Esterbauer et al., 1991), it can indicate ROS-related plant stress and lipid injury (Davey et al., 2005). In the present research, MDA was unaffected by any R:B or FR combination (**Supplementary Figure 2C**), indicating that the ROS-scavenging abilities of these antioxidant compounds were sufficient to maintain lasting ROS-related damage to consistent and manageable levels. Finally, in this study, phenolic compound concentration (**Supplementary Figure 2B**) was lower than total flavonoid concentration (**Figure 4A**), an anomaly that is likely inaccurate as flavonoids are a subclass of phenolic compounds. This inaccuracy is prospectively due to different extraction methods and quantification with different standards; therefore, total phenolic compound concentrations should be considered relative and only be compared to each other for trends based on B content and FR presence.

Other pigments, chlorophyll and carotenoids, also increased as R:B decreased (**Figure 2**). Carotenoids have photoprotective mechanisms, controlling the energy flux to chlorophylls and managing oxidative stress. In lettuce, the two most common carotenoids are lutein and β-carotene, other group members include zeaxanthin, violaxanthin, astaxanthin, and lactucaxanthin (Phillip and Young, 1995; Mou, 2009; Kim et al., 2016; Yang et al., 2022). Although this study did not explore individual carotenoids, it would be prudent to further explore these individual carotenoids. This would determine if all carotenoid production is induced under high blue irradiation, or if specific carotenoids are induced. Additionally, some carotenoids convert to other carotenoids as a stress response to changes in light intensity (Sajilata et al., 2008). Therefore, it is probable that oxidative stress from higher-energy blue light may induce similar types of carotenoid conversion. There have been reports of increased B content resulting in increased carotenoids in several plant species and microalgae (Samuolienė et al., 2017; Zhang et al., 2022), but B content-induced carotenoid interconversion has yet to be explored in detail. This can further be expanded by including FR, as we found a novel interactive effect between FR and B on carotenoid concentration, where B light caused greater carotenoid content with added FR (**Figure 2B**).

### 4.3 Carbohydrates increase from FR addition, decrease with increased B content

We analyzed carbohydrates because they are nutritional and energy sources for plants and humans (Apriyanto et al., 2022). Here, carbohydrate concentration decreased with B content but increased with FR addition, but the two spectra had no interaction. This pattern was similar to the measurements of shoot fresh weight and leaf area, likely because carbohydrate pools are tightly associated with plant growth. Plant carbohydrates are classified as structural or non-structural, either contributing to cell wall and plant stem structural components (Martínez-Vilalta et al., 2016; Tarasov et al., 2018), or steering plant metabolism as sources of energy resulting from photosynthesis (Bolton, 2009; Rosa et al., 2009; Apriyanto et al., 2022). This energy primarily is in the form of soluble sugars or starch, a storage carbohydrate that can be metabolized to provide plant organs with carbon and energy (Zeeman et al., 2010; Apriyanto et al., 2022). Soluble sugars are the accessible form of this energy, but starch contributes to plant growth, protection, improving tolerance to drought, temperature, and salinity stress (Rosa et al., 2009; Krasavina et al., 2014). The reduction of carbohydrates under low R:B is possibly a result of a shift in prioritizing energy resources towards specialized metabolite synthesis, as was seen in this study with increased antioxidant compound accumulation. Such a transition to focus on specialized metabolism over growth may occur in plants experiencing environmental stressors including light stresses, drought, and temperature stress (Dixon and Paiva, 1995; Seigler, 1998; Qaderi et al., 2023). Intriguingly, we found that FR restores the decrease in carbohydrates due to low R:B, also improving or equaling the highest carbohydrate concentration of plants grown without FR. Elevated carbohydrate content with FR confirms previous research (Yang et al., 2016; Courbier et al., 2020) and may improve consumer perceptions by increasing lettuce shelf life, sweetness, and crispness (Witkowska and Woltering, 2010; Lin et al., 2013; Min et al., 2021). Hence, growers will find it attractive to use FR to simultaneously improve carbohydrate content and crop growth.

### 4.4 Macronutrients and micronutrients

Finally, we analyzed mineral content because humans require a balanced intake of minerals for their health and metabolism. While blue light has been shown to increase the concentration of common nutritional minerals in crops such as broccoli and lettuce, effects differ depending on the mineral of interest (Kopsell et al., 2014; Lee et al., 2019). Most of the analyzed macro- and micronutrient concentrations in the present study were unaffected by FR addition, with FR only significantly decreasing the concentration of calcium (reduced with FR addition from high R:B to low R:B by 3-9%), phosphorus (9-27%), magnesium (5-15%), and manganese (13-25%) (**Figure 5**, **Table 3**). There was no interaction between FR and B content found to affect any mineral concentrations, but a higher percentage of blue light in an R:B spectrum did increase the mineral concentrations for most elements (ranging from 7-26%), with the exception of boron, chloride, iron, and magnesium (**Figure 5**, **Table 3**). While intriguing that increases with B content occurred for most nutrients, these changes in mineral concentrations should be put into perspective with biologically relevant changes in mineral concentration. Although there were significantly different changes in mineral concentration, each was within range of commonly found lettuce nutrient concentrations (Hartz et al., 2007). This being said, we also saw that when considering the macro- and micronutrient content in terms of micro- or milligrams per plant (**Supplementary Table 2**), there was a significant effect of FR for each nutrient. This is logical, as the plants grown under FR light had a much larger size, resulting in more total nutrients per plant, while still having a reduced concentration per gram of fresh weight. However, we propose that the nutrient concentration per gram is more valuable as a nutritional aspect for equal portions of food, as concentration is more of a determinant of the leaf tissue nutritional potency. The effect of R:B and FR on mineral and nutritional content requires further investigation, as some nutrients seem to be enhanced by altering R:B ratio, while others by FR, and others don’t appear to be affected by either. Finally, as nitrates are an important factor for human health considerations, it is prudent to mention that the nitrate concentration of lettuce in this study (~1250 – 2250 mg/kg fresh weight) was well below the 5000 mg/kg maximal limitation set in place by the EU (Commission Regulation (EU) No. 1258/2011).

### 4.5 The effects of blue light and FR radiation in an R:B spectrum were additive

In a previous study, Meng and Runkle (2019) found that additional FR radiation antagonized blue radiation effects on growth in an R:B background. In the present study, many effects of FR were equally affected with each corresponding increase in B content, meaning individual effects were more or less additive. The discrepancy here may be because young lettuce seedlings were the focus in Meng and Runkle (2019), whereas the present study analyzed lettuce grown to a harvestable and nutritionally relevant stage. Therefore, early growth stages may have interactive effects from R:B and FR, however over the course of development, these effects transition to be additive. This may also indicate that the light response pathways and triggered regulatory genes for growth and nutritional compounds in lettuce have more specific regulatory patterns.

Very high fractions of blue light may change the Pr/Pfr ratio (Hogewoning et al., 2010). However, the values of PSS, which can be considered as an estimate for this ratio (Sager et al., 1988), did not vary a lot among the R:B treatments applied in this research, while the PSS values by the additional FR were reduced from 0.87-0.88 to 0.78-0.82 (**Table 1**). This, combined with the determination that there were individual responses to R:B and FR treatments, we postulate that the plant responses to FR act via phytochromes independently from the response to R:B acting via B light photoreceptors. This may be further supported considering the results of the reference white light treatments. White light (with and without FR) had very similar PSS values to the closest corresponding R:B ratio (R:B_87.5:12.5_, with or without FR), and nearly every measured parameter under white light was not significantly different from those of the nearest corresponding R:B ratio. Therefore, morphology, metabolites, and minerals were more greatly affected by the B content, FR addition, or both together, rather than the presence of green-yellow light or small changes to PSS value.

Because of the additive effects of FR and R:B ratios, their individual benefits can be harnessed by utilizing combined spectra applications to cooperatively benefit both growth and nutrition. By designing growth recipes considering both yield and nutritional quality, growers can improve produce for end consumers by producing more nutritional crops in greater size or number. Growers do not directly benefit from plants’ nutritional contents – consumers consume the crops. However, nutritional quality is largely recognized by consumers, boosting or reducing sales of growers’ crop. Conversely, consumers, often unaware of their purchased vegetables’ growth cycles, are nonetheless affected by food shortages due to long cultivation periods. This duality of recognizing the primary desires of both parties, should also be considered by both parties. Ultimately, it falls to growers to address each aspect during cultivation; both yield and nutritional quality should be considered and valued throughout the lifetime of a crop, from sowing to consumption.

### 4.6 Increased B content decreases energy-use efficiency, whereas FR increases efficiency

Although LEDs are overall more efficient than other lighting technologies like fluorescent or high-pressure sodium lights (Pennisi et al., 2019b; Neo et al., 2022), there are differences in efficacy of LEDs producing different wavelengths (Kusuma et al., 2022). Of the studied wavelengths, at present, B LEDs have the lowest efficacy, followed by FR, then R, which has the highest efficacy of LED-produced wavelengths (Kusuma et al., 2022). Simply, high B content in a growth recipe often results in a lower efficiency than a growth recipe with lower B, which has previously been described in the growth of tomato plants (Kusuma et al., 2023). Consequently, a balance is required while improving plant growth or nutritional content, considering radiation- or energy-use efficiency. Here, the addition of FR had a significant increase in radiation- and energy-use efficiency for all spectra (**Figure 6**), confirming previous studies in lettuce (Jin et al., 2021). That is, although the total light and resulting electricity usage were increased, plants were able to utilize light energy more efficiently, ultimately producing greater biomass per photon or kilowatt of energy. Furthermore, we found that the negative effect of increased B content was ameliorated when FR was added to the spectra (**Figure 6**). Therefore, the enhanced production of metabolites under high B can also be harnessed using this improved efficiency with FR inclusion. Importantly, we recognize that the presented values of radiation- and energy-use efficiency were overall relatively low, which is due to the low planting density of this study; high planting densities have previously been found to dramatically increase both radiation- and energy-use efficiencies (Jin et al., 2021). The efficiency values reported here can easily be increased by growing plants in more dense arrangements, as the plants in this study had ample room for growth.

### 4.7 Considerations and future directions

Some considerations for this study are important to note. First, although FR addition on average reduced metabolite and nutrient concentrations, these values were calculated on the basis of per gram of fresh lettuce tissue (or per gram dried lettuce tissue as for the analyzed macro- and micro-elements). Far-red application resulted in larger plants, therefore the total amount of nutrients per plant (instead of per unit fresh or dry weight) could be even greater with added FR, however with less potency than the R:B counterparts. Secondly, as previously mentioned, this work correlates the analyzed parameters with the content of B light in an R:B spectrum, so with an increase of B, there is a corresponding decrease of R. Therefore, responses may be due to increased B content, decreased R content, or both. This may require further analysis, potentially by replacing R or B with another wavelength (e.g. green light) to determine monochromatic ratio effects. Finally, the estimated phytochrome photostationary state (PSS) value is slightly different for the R:B+FR treatments in this study, due to their different spectral compositions (**Table 1**). PSS is the ratio of Pfr (i.e. the active form of phytochrome) to the total phytochrome and represents the amount of phytochrome that can perform physiological responses (Kreslavski et al., 2018). This may indicate that the results analyzed when considering FR may be due to the general supplementation of FR, the changed R:FR, or PSS value.

This study’s methodology used light treatments of FR with R:B ratios throughout cultivation to present the advantages of spectral growth conditions primarily in two directions. One direction (FR addition) improves carbohydrates and yield, while the other direction (high B in an R:B background) holds more potency in improved nutritional quality. Consequently, CEA can utilize light treatments that capitalize on the benefits of multiple wavelengths. By performing customized recipes there may be an approach to have the best of both worlds, maximizing yield and nutritional quality.

## 5 Conclusions

In this study, we described that far-red and red:blue ratios affect plant growth and nutritional quality in an additive manner. Higher amounts of blue light in a red:blue background improved the concentrations of antioxidant metabolites and certain nutrients in lettuce, compounds which are associated with elevated nutritional value. When supplemental far-red was added to any red:blue background, lettuce consistently had improved growth and carbohydrate concentration compared to the red:blue backgrounds without far-red. Specifically, a low red:blue ratio, when combined with supplemental far-red, was most successful at maintaining growth (or limiting the negative growth effects of a low red:blue ratio without far-red). Importantly, lettuce growth under low red:blue ratios with supplemental far-red light also accumulated greater concentrations of (non-)photosynthetic pigments, sugars, starch, and certain key nutrients. Lastly, this study was designed using treatments that can feasibly be implemented into a controlled environment agriculture system with little modulation necessary, adjustable based on desired growth or nutritional preferences. Future studies should further analyze red:blue and far-red interactions on the production of these, and other, nutritional compounds via -omics studies to further improve the growing repertoire of knowledge on plant production in controlled environment agriculture.

## Data availability statement

The original contributions presented in the study are included in the article/**Supplementary Material**. Further inquiries can be directed to the corresponding author.

## Author contributions

JB: Data curation, Formal analysis, Investigation, Methodology, Validation, Visualization, Writing – original draft, Writing – review & editing. SC: Conceptualization, Investigation, Methodology, Supervision, Writing – review & editing, Data curation, Formal analysis, Validation, Visualization. CK: Data curation, Formal analysis, Investigation, Methodology, Writing – original draft, Visualization. JV: Supervision, Writing – review & editing. LM: Funding acquisition, Project administration, Supervision, Writing – review & editing.
