# Corrigendum: Paradise by the far-red light: Far-red and red:blue ratios independently affect yield, pigments, and carbohydrate production in lettuce, *Lactuca sativa*

**DOI:** 10.3389/fpls.2024.1442349

**Published:** 2024-06-18

**Authors:** Jordan B. Van Brenk, Sarah Courbier, Celestin L. Kleijweg, Julian C. Verdonk, Leo F. M. Marcelis

**Affiliations:** ^1^ Horticulture and Product Physiology, Plant Sciences Group, Wageningen University and Research, Wageningen, Netherlands; ^2^ Faculty of Biology II, University of Freiburg, Freiburg, Germany; ^3^ Centre for Integrative Biological Signalling Studies (CIBSS), University of Freiburg, Freiburg, Germany

**Keywords:** controlled environment agriculture, light quality, far-red light, red:blue ratio, nutritional quality, metabolic compounds, product physiology

**Text Correction**

In the published article, there was an error. In the **Abstract**, the word “Increasing” was used instead of “Decreasing” in the following sentence: “Increasing the R:B ratio from R:B_87.5:12.5_ to R:B_60:40_, there was a decrease in fresh weight (20%) and carbohydrate concentration (48% reduction in both sugars and starch), whereas pigment concentrations (anthocyanins, chlorophyll, and carotenoids), phenolic compounds, and various minerals all increased.”

A correction has been made to the **Abstract**. This sentence previously stated:

“Increasing the R:B ratio from R:B_87.5:12.5_ to R:B_60:40_, there was a decrease in fresh weight (20%) and carbohydrate concentration (48% reduction in both sugars and starch), whereas pigment concentrations (anthocyanins, chlorophyll, and carotenoids), phenolic compounds, and various minerals all increased.”

The corrected sentence appears below:

“Decreasing the R:B ratio from R:B_87.5:12.5_ to R:B_60:40_, there was a decrease in fresh weight (20%) and carbohydrate concentration (48% reduction in both sugars and starch), whereas pigment concentrations (anthocyanins, chlorophyll, and carotenoids), phenolic compounds, and various minerals all increased. ”

The authors apologize for this error and state that this does not change the scientific conclusions of the article in any way. The original article has been updated.

