# Supplementary material for: Paradise by the far-red light: Far-red and red:blue ratios independently affect yield, pigments, and carbohydrate production in lettuce, Lactuca sativa
Source: Front Plant Sci. 2024 Apr 30;15:1383100. doi: 10.3389/fpls.2024.1383100 (PMC11091871; doi:10.3389/fpls.2024.1383100)
Supplement: Supplementary file 1 [file DataSheet_1.docx]

Supplementary Material

# Supplementary Data

**A**

**B**

**D**


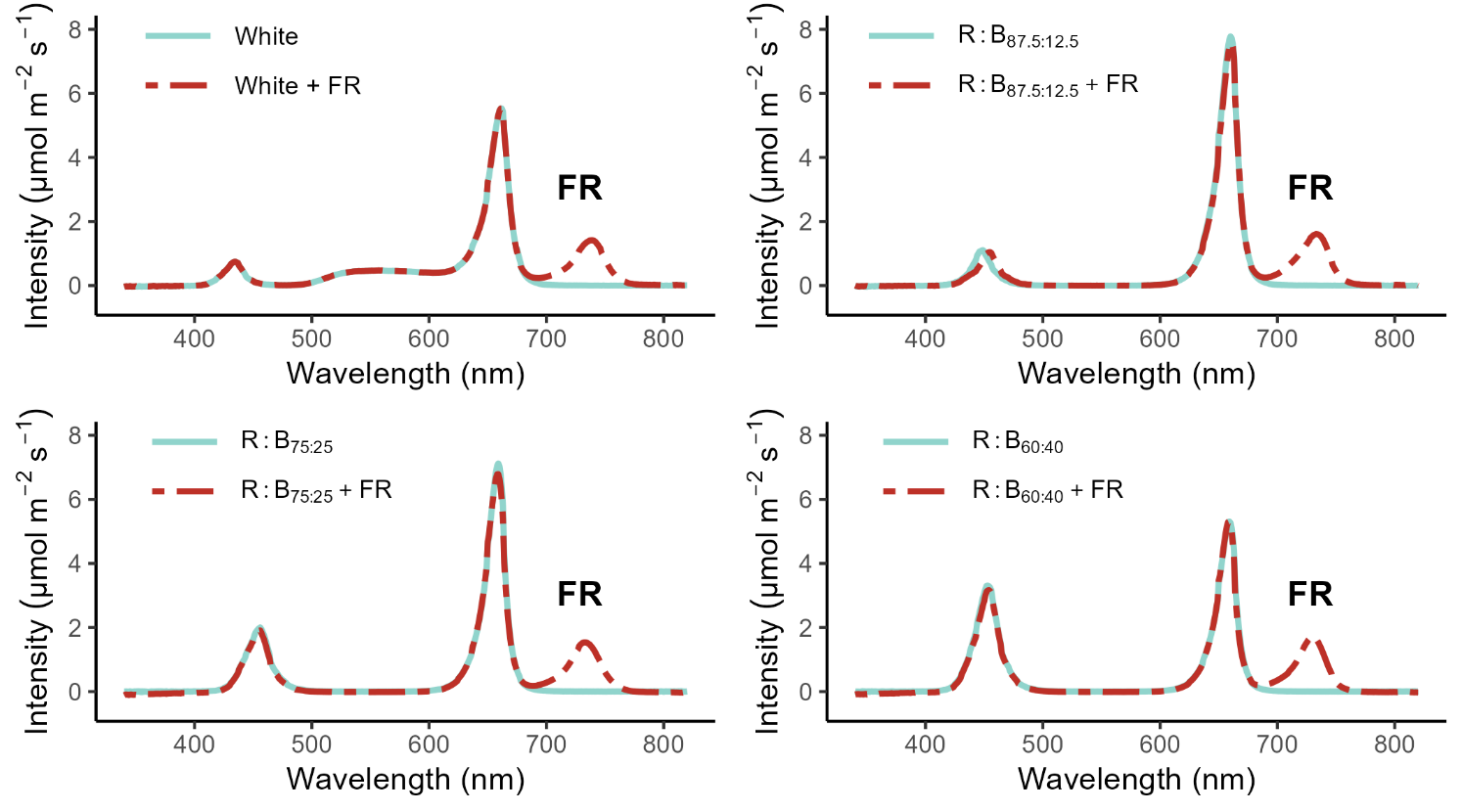


**C**

**Supplementary Figure 1. Treatment spectral compositions**

Spectral compositions of eight 200 µmol m^-2^ s^-1^ PAR light treatments. The treatments consist of: (**A**) white light and three red:blue ratios: (**B**) R:B_87.5:12.5_, (**C**) R:B_75:25_, and (**D**) R:B_60:40_. Each of these four treatments either had 50 µmol m^-2^ s^-1^ of supplemental far red light (indicated by +FR), or no supplemental far red.


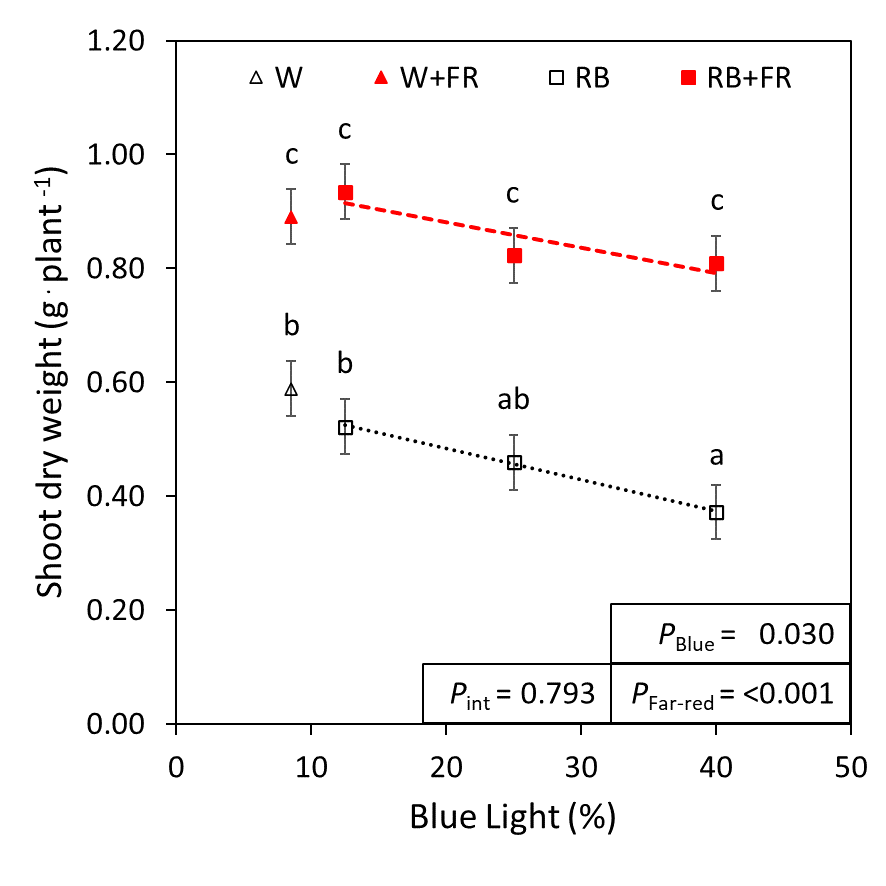

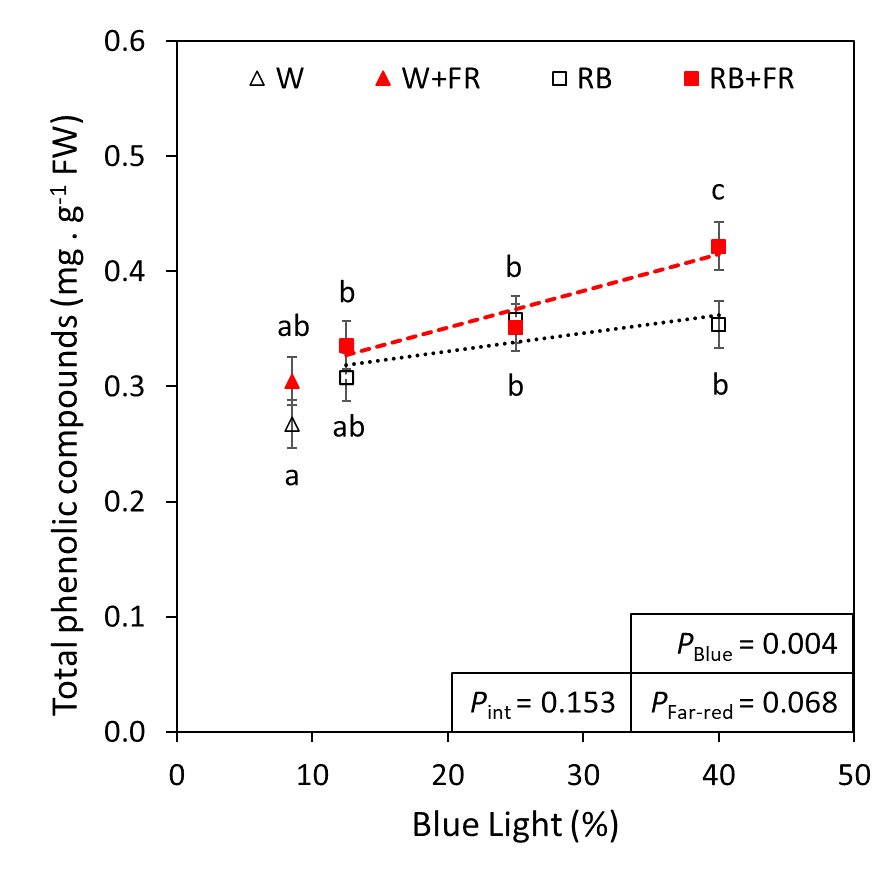

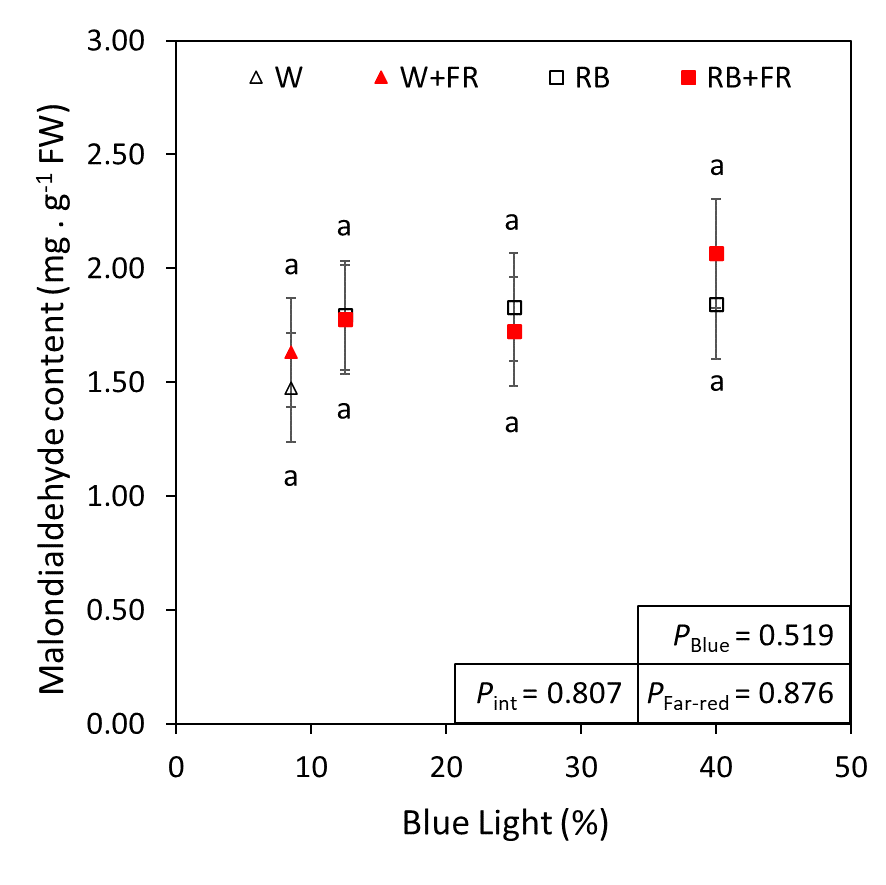


**C**

**B**

**A**

**Supplementary Figure 2. Shoot dry weight, phenolic compounds, and malondialdehyde content of lettuce grown under red:blue ratios with or without supplemental far-red.**

Shoot dry weight weight (**A**), total phenolic content (**B**), and malondialdehyde content of lettuce grown under different red:blue ratios presented by blue light % in the R:B spectrum (R:B_87.5:12.5_ = 12.5% blue; R:B_75:25_ = 25% blue; and R:B_60:40_ = 40% blue), with additional far-red light (RB+FR) or no far-red (RB). White light (~8.5% blue) is used as a comparison, with far-red (W+FR) or without (W). Trendlines were drawn to indicate the probability of a linear relationship with blue light (*P*_Blue_, α = 0.05). Different letters indicate significantly different values for each combination of R:B ratio and FR light treatments, according to an unprotected Fisher LSD Test (α = 0.05). Datapoints represent treatment means with error bars representing standard error means of three growth cycles (*n* = 3), each consisting of ten or four replicate plants. *P*_Far-red_ = probability of an effect from far-red, *P*_int_ = probability of an interactive effect between blue content and far-red addition.


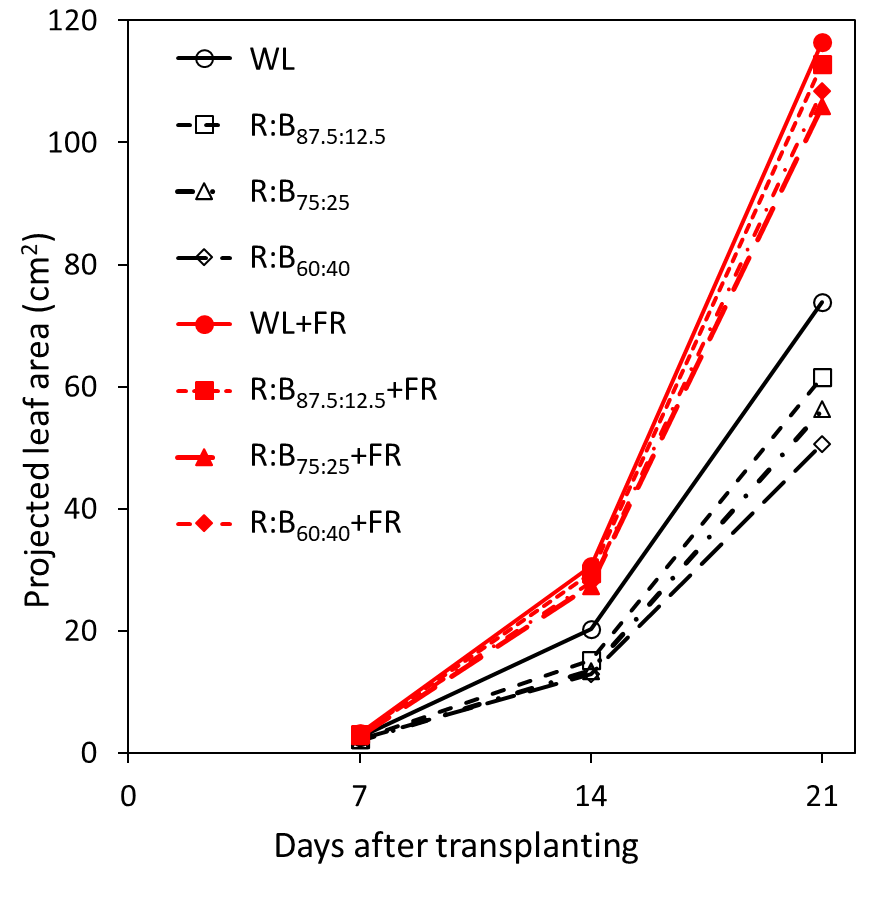


**Supplementary Figure 3. Projected leaf area of different red:blue ratios with or without far red application.**

The projected leaf area of lettuce grown under three different red:blue light ratios (R:B_87.5:12.5_, R:B_75:25_, R:B_60:40_) with additional far-red light (RB+FR) or no far-red (RB). White light (~8.5% blue) is used as a comparison, with far-red (WL+FR) or without (WL). Datapoints represent treatment means of three growth cycles (*n* = 3), each consisting of nine to ten replicate plants.

| Parameter | **FR** | **W** | **R:B ^87.5:12.5^** | **R:B ^75:25^** | **R:B ^60:40^** | **SEM^ǂ^** | ***P*_Blue_^ǂǂ^** | ***P*_Far-red_^ǂǂǂ^** |
| --- | --- | --- | --- | --- | --- | --- | --- | --- |
| Chlorophyll a  (mg g^-1^ FW) | No FR | 0.3712^a^ | 0.3940^ab^ | 0.4526^bc^ | 0.4613^c^ | ± 0.02018 | <0.001^*^ | 0.066 |
|  | +FR | 0.3605^a^ | 0.3714^a^ | 0.3750^a^ | 0.4739^c^ |  |  |  |
| Chlorophyll b  (mg g^-1^ FW) | No FR | 0.1708^a^ | 0.1737^ab^ | 0.1996^bc^ | 0.2096^c^ | ± 0.0088 | <0.001^*^ | 0.184 |
|  | +FR | 0.1647^a^ | 0.1644^a^ | 0.1718^a^ | 0.2185^c^ |  |  |  |
| $\frac{Chl a}{Chl b}$ | No FR | 2.1464^ab^ | 2.2312^d^ | 2.2272^cd^ | 2.1641^abc^ | ±0.02094 | <0.001^*^ | <0.001^*^ |
|  | +FR | 2.1659^abc^ | 2.2026^bcd^ | 2.1217^a^ | 2.1303^a^ |  |  |  |
| $\frac{Total Chl}{\mathrm{Carotenoids}}$ | No FR | 7.257^c^ | 6.755^abc^ | 6.669^ab^ | 7.022^bc^ | ± 0.1703 | 0.063 | 0.021^*^ |
|  | +FR | 6.807^abc^ | 6.423^a^ | 6.378^a^ | 6.711^ab^ |  |  |  |

**Supplementary Table 1. Pigments in lettuce grown under different light spectra.**

Note: W = white light; R:B_87.5:12.5_, R:B_75:25_, R:B_60:40_ = R:B ratios used in this study; FR = supplemental far-red light; FW = Fresh weight; Chl = chlorophyll.

**^ǂ^** SEM, standard error means of three growth cycles (*n* = 3) each consisting of eight replicate plants for all eight light treatments. Different letters indicate significantly different values for each combination of R:B ratio and FR light treatments, using an unprotected Fisher LSD Test (α = 0.05).

**^ǂǂ^** P-value for blue content effects among the three levels of blue light according to a two-way ANOVA.

**^ǂǂǂ^** P-value for far-red light effects among the three levels of blue light according to a two-way ANOVA.

^*^ Denotes a significant effect of either *P*_Blue_ or *P*_Far-red_ (α = 0.05).

| Element | **FR** | **W** | **R:B ^87.5:12.5^** | **R:B ^75:25^** | **R:B ^60:40^** | **SEM^ǂ^** | ***P*_Blue_^ǂǂ^** | ***P*_Far-red_^ǂǂǂ^** | ***P*_int_^ǂǂǂǂ^** |
| --- | --- | --- | --- | --- | --- | --- | --- | --- | --- |
| Boron  (µg plant^-1^) | No FR | 15.60^c^ | 13.09^bc^ | 11.01^ab^ | 9.320^a^ | ±0.776 | 0.003^*^ | <0.001^*^ | 0.046^*^ |
|  | +FR | 23.71^e^ | 21.78^de^ | 19.54^de^ | 22.40^e^ |  |  |  |  |
| Calcium | No FR | 6.375^c^ | 5.668^bc^ | 5.042^ab^ | 4.185^a^ | ±0.320 | 0.005^*^ | <0.001^*^ | 0.060 |
| (mg plant^-1^) | +FR | 9.852^e^ | 9.277^e^ | 7.939^d^ | 9.206^e^ |  |  |  |  |
| Chloride  (mg plant^-1^) | No FR | 4.714^c^ | 4.157^bc^ | 3.527^ab^ | 2.844^a^ | ±0.320 | 0.011^*^ | <0.001^*^ | 0.392 |
|  | +FR | 7.514^e^ | 7.087^de^ | 6.259^d^ | 6.622^de^ |  |  |  |  |
| Copper  (µg plant^-1^) | No FR | 5.045^ab^ | 4.298^a^ | 4.323^a^ | 3.764^c^ | ±0.496 | 0.305 | <0.001^*^ | 0.195 |
|  | +FR | 7.764^c^ | 6.892^c^ | 6.633^bc^ | 8.287^c^ |  |  |  |  |
| Iron  (µg plant^-1^) | No FR | 106.6^abc^ | 80.6^ab^ | 122.0^abcd^ | 74.50^a^ | ±18.06 | 0.734 | 0.002^*^ | 0.224 |
|  | +FR | 136.6^bcd^ | 157.2^cd^ | 153.9^cd^ | 175.5^d^ |  |  |  |  |
| Magnesium  (mg plant^-1^) | No FR | 1.580^b^ | 1.450^b^ | 1.308^ab^ | 1.091^a^ | ±0.093 | 0.016^*^ | <0.001^*^ | 0.129 |
|  | +FR | 2.446^d^ | 2.287^d^ | 1.967^c^ | 2.268^cd^ |  |  |  |  |
| Manganese  (µg plant^-1^) | No FR | 24.11^bc^ | 19.17^ab^ | 18.86^ab^ | 16.66^a^ | ±1.602 | 0.038^*^ | <0.001^*^ | 0.275 |
|  | +FR | 32.09^e^ | 29.15^cde^ | 25.83^cd^ | 30.11^de^ |  |  |  |  |
| Molybdenum  (µg plant^-1^) | No FR | 0.539^abc^ | 0.456^ab^ | 0.428^ab^ | 0.371^a^ | ±0.072 | 0.090 | <0.001^*^ | 0.041^*^ |
|  | +FR | 0.911^de^ | 0.777^cd^ | 0.668^bc^ | 1.116^e^ |  |  |  |  |
| Nitrate | No FR | 20.93^abc^ | 17.77^ab^ | 16.61^a^ | 16.24^a^ | ±3.270 | 0.155 | <0.001^*^ | 0.238 |
| (mg plant^-1^) | +FR | 38.59^de^ | 31.70^cde^ | 27.74^bcd^ | 41.60^e^ |  |  |  |  |
| Nitrogen  (mg plant^-1^) | No FR | 31.36^b^ | 27.46^ab^ | 24.27^ab^ | 21.33^a^ | ±2.469 | 0.052 | <0.001^*^ | 0.242 |
|  | +FR | 50.12^d^ | 44.81^cd^ | 40.17^c^ | 47.63^cd^ |  |  |  |  |
| Phosphorus | No FR | 4.007^bc^ | 3.389^abc^ | 3.289^ab^ | 2.890^a^ | ±0.270 | 0.018^*^ | <0.001^*^ | 0.162 |
| (mg plant^-1^) | +FR | 5.916^d^ | 5.276^d^ | 4.285^c^ | 5.285^d^ |  |  |  |  |
| Potassium | No FR | 51.86^c^ | 44.46^bc^ | 39.27^ab^ | 32.89^a^ | ±2.850 | 0.009^*^ | <0.001^*^ | 0.036^*^ |
| (mg plant^-1^) | +FR | 81.68^e^ | 74.52^de^ | 66.15^d^ | 79.62^e^ |  |  |  |  |
| Sodium  (mg plant^-1^) | No FR | 0.471^a^ | 0.399^a^ | 0.406^a^ | 0.371^a^ | ±0.053 | 0.522 | <0.001^*^ | 0.625 |
|  | +FR | 0.773^b^ | 0.687^b^ | 0.715^b^ | 0.788^b^ |  |  |  |  |
| Sulfur  (mg plant^-1^) | No FR | 1.849^b^ | 1.625^ab^ | 1.450^a^ | 1.274^a^ | ±0.113 | 0.035^*^ | <0.001^*^ | 0.038^*^ |
|  | +FR | 2.912^d^ | 2.676^cd^ | 2.431^c^ | 3.008^d^ |  |  |  |  |
| Zinc  (µg plant^-1^) | No FR | 16.16^ab^ | 12.53^a^ | 12.60^a^ | 11.20^a^ | ±1.661 | 0.175 | <0.001^*^ | 0.116 |
|  | +FR | 25.03^cd^ | 23.11^cd^ | 20.86^bc^ | 28.11^d^ |  |  |  |  |

Supplementary Table 2. Macro- and micro-elements per plant in lettuce grown under different light spectra.

Note: W = white light; R:B_87.5:12.5_, R:B_75:25_, R:B_60:40_ = R:B ratios used in this study; FR = supplemental far-red light; DW = dry weight.

**^ǂ^** SEM, standard error means of two growth cycles (*n* = 2), consisting of multiple plants (10 to 26, depending on treatment) for all eight light treatments. Different letters indicate significantly different values for each combination of R:B ratio and FR light treatments, using an unprotected Fisher LSD Test (α = 0.05).

**^ǂǂ^** P-value for blue content effects among the three levels of blue light according to a two-way ANOVA.

**^ǂǂǂ^** P-value for far-red light effects among the three levels of blue light according to a two-way ANOVA.

**^ǂǂǂǂ^** P-value for interactive effects between far-red and blue light according to a two-way ANOVA.

^*^ Denotes a significant effect of either *P*_Blue_ or *P*_Far-red_ (α = 0.05).
